# Sex differences in the case-fatality rates for COVID-19—A comparison of the age-related differences and consistency over seven countries

**DOI:** 10.1371/journal.pone.0250523

**Published:** 2021-04-29

**Authors:** Manfred S. Green, Dorit Nitzan, Naama Schwartz, Yaron Niv, Victoria Peer

**Affiliations:** 1 School of Public Health, University of Haifa, Haifa, Israel; 2 World Health Organization, European Region, Copenhagen, Denmark; 3 Israel Ministry of Health, Jerusalem, Israel; Università degli Studi di Ferrara, ITALY

## Abstract

**Background:**

Early in the COVID-19 pandemic, it was noted that males seemed to have higher case-fatality rates than females. We examined the magnitude and consistency of the sex differences in age-specific case-fatality rates (CFRs) in seven countries.

**Methods:**

Data on the cases and deaths from COVID-19, by sex and age group, were extracted from the national official agencies from Denmark, England, Israel, Italy, Spain, Canada and Mexico. Age-specific CFRs were computed for males and females separately. The ratio of the male to female CFRs were computed and meta-analytic methods were used to obtained pooled estimates of the male to female ratio of the CFRs over the seven countries, for all age-groups. Meta-regression and sensitivity analysis were conducted to evaluate the age and country contribution to differences.

**Results:**

The CFRs were consistently higher in males at all ages. The pooled M:F CFR ratios were 1.71, 1.88, 2.11, 2.11, 1.84, 1.78 and 1.49, for ages 20–29, 30–39, 40–49, 50–59, 60–69, 70–79, 80+ respectively. In meta-regression, age group and country were associated with the heterogeneity in the CFR ratios.

**Conclusions:**

The sex differences in the age-specific CFRs are intriguing. Sex differences in the incidence and mortality have been found in many infectious diseases. For COVID-19, factors such as sex differences in the prevalence of underlying diseases may play a part in the CFR differences. However, the consistently greater case-fatality rates in males at all ages suggests that sex-related factors impact on the natural history of the disease. This could provide important clues as to the mechanisms underlying the severity of COVID-19 in some patients.

## Introduction

Early in the course of the COVID-19 pandemic, it was observed that there were sex differences in the incidence of the disease [1]. However, this observation has been inconsistent and it is possible that some of the differences observed were due to differences in exposure [2, 3]. In some countries, many of the cases were healthcare workers, who may be overrepresented by females [2, 3]. In South Korea, a large outbreak of COVID-19 occurred in a religious group whose members were predominantly young women [4]. While incidence rate is directly related to exposure, the case-fatality proportion, which is the proportion of deaths among the cases during a specified period of time is a measure of the severity of the disease. It is commonly referred to as the case-fatality rate (CFR) [5–7] and has been reported as being higher in males than females [8]. Since the CFR does not contain any measure of the population exposed, it is less likely to be affected by exposure of the cases [9, 10].

Evidence of sex differences in the CFR for COVID-19, could provide clues to the mechanisms of the infection [11]. Sex differences have been documented for a number of infectious diseases. In general, males tend to have higher incidence rates for many infectious diseases, although not in all age groups [12, 13]. Pertussis is a notable exception, where females dominate in the incidence rates in most age groups [14]. Regarding sex differences in mortality, in a nationwide study of infectious diseases mortality rates in the United States in 1980, prior to the HIV/AIDS epidemic, the rates in males were about 50% higher than in females [15]. The leading cause of infectious disease mortality was from respiratory diseases [15]. The exact mechanism of the sex differences in the incidence and mortality from infectious diseases is not well-understood [11]. Elucidation of the sex differences in the COVID-19 CFR could contribute to a better understanding of the pathogenesis of COVID-19. In this paper we used meta-analytic methods to examine the magnitude and consistency of sex differences in the COVID-19 CFRs by age group, in seven countries.

## Materials and methods

### Source of data and search strategy

**Fig 1:**
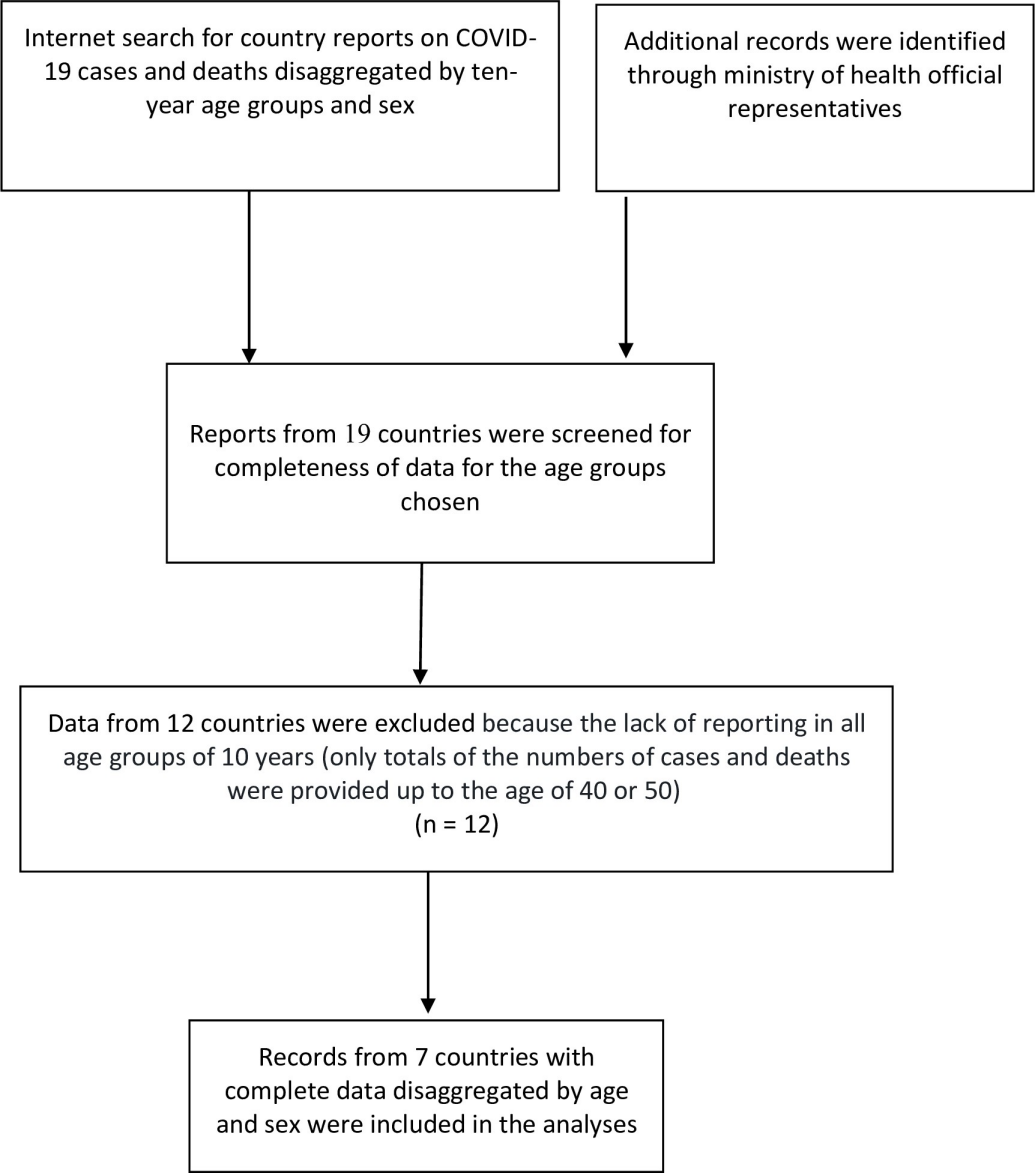
PRISMA flowchart describing the included and excluded data.

This was a comparative study of retrospective national cohorts of COVID-19. We searched the internet and national health ministry sites for data on COVID-19 cases and deaths, disaggregated by age group and sex. Although this was not a classical systematic review, we used the PRISMA 2009 checklist for guidance in searching for complete national databases (Fig 1).

We were able to locate complete data with comparable age intervals from official published reports for seven countries: Canada, Denmark, England, Israel, Italy, Mexico and Spain. The number of deaths in each age group and sex for Denmark, England and Spain were obtained from official source [16]. The number of confirmed cases of COVID-19 by age group and sex were available for Denmark (for February 2020—December 2020) from Statista [17] and from the official institutes of England [18] and Italy [19] for January 2020- February 2021. The number of confirmed cases and deaths on COVID-19 in Spain, between January 2020 and February 2021, were available from Instituto de Salud Carlos III [20]. Data on COVID-19 cases and death by age group and sex were extracted from the official websites of the government of Canada for January 2020- February 2021 [21], and the governments of Italy [19] and Mexico from February 2020 to February 2021 [22]. The data on COVID-19 for Israel from February 2020 to December 2020 were obtained directly from the Ministry of Health.

### Ethical considerations and informed consent

National, open access aggregative and anonymous data were used and there was no need for ethics committee approval.

### Statistical analyses

The COVID-19 CFRs by sex and age group were defined as the number of deaths divided by the number of reported cases in each respective sub-group. The age groups were divided by intervals: 0–19, 20–29, 30–39, 40–49, 50–59, 60–69, 70–79, 80+. The ratio of the male to female CFRs (CFRR) was calculated by dividing the CFRs for males by the CFRs for females, by age group and country. We used meta-analytic methodology to evaluate the overall magnitude and consistency of the sex differences in the CFR of COVID-19 by age group, across different countries. The outcome variable was the male to female CFRR. The independent variables were age group and country. The data presented (forest plot) are the CFRRs by age group and country. Heterogeneity was evaluated using Cochran’s Q statistic. Tau^2^ and I^2^ were used to estimate the between-group variance. If the Q test yielded a p < 0.1, and/or I^2^≥50%, the random effects model [23] was used to estimate pooled CFRRs and 95% confidence intervals (CI). Otherwise the fixed-effects model was used. To evaluate the effect of individual county on the risk of COVID-19, we performed leave-one-out sensitivity analysis and recomputed the pooled CFRRs. WE further explored the associations of the CFRRs with age-group and country using meta-regression analyses. The meta-analytic methods were carried out using STATA software version 12.1 (Stata Corp., College Station, TX).

## Results

The summary of male and female CFRRs in different countries for each age group is presented in Table 1.

**Table 1:** Cases, deaths, CFRs (%) (by age group and sex) and male to female CFRRs (%).

Age Group	Countries	Males cases	Males death	Male CFR	Females cases	Females death	Female CFR	Male: Female CFRR
0–9	Italy	61722	4	0.01	57271	6	0.01	0.62
	Denmark	3637	*N/A	*N/A	3399	*N/A	*N/A	*N/A
	England	79886	2	0.00	75681	4	0.01	0.47
	Israel	26690	3	0.01	25221	1	0.00	
	Spain	504	1	0.20	435	1	0.23	0.86
	Canada	**no data	**no data	**no data	**no data	**no data	**no data	**no data
	Mexico	11228	153	1.36	10039	129	1.28	1.06
10–19	Italy	123123	5	0.00	113091	5	0.00	0.00
	Denmark	10975	*N/A	*N/A	10165	*N/A	*N/A	*N/A
	England	168844	15	0.01	190305	10	0.01	1.69
	Israel	45191	0	0	34289	2	0.01	0
	Spain	806	3	0.37	955	2	0.21	1.78
	Canada	**no data	**no data	**no data	**no data	**no data	**no data	**no data
	Mexico	37819	82	0.22	39642	99	0.25	0.87
20–29	Italy	163197	29	0.02	161123	20	0.01	1.43
	Denmark	12158	*N/A	*N/A	*N/A	*N/A	*N/A	*N/A
	England	289285	88	0.03	354648	56	0.02	1.93
	Israel	42443	4	0.01	41521	4	0.01	0.98
	Spain	278302	59	0.02	301370	46	0.02	1.39
	Canada	78252	19	0.02	76271	14	0.02	1.32
	Mexico	165910	1170	0.71	178436	726	0.41	1.73
30–39	Italy	164200	109	0.07	172681	69	0.04	1.66
	Denmark	8287	1	0.01	8508	2	0.02	0.51
	England	282615	296	0.10	334428	207	0.06	1.69
	Israel	29240	11	0.04	29997	7	0.02	1.61
	Spain	196390	102	0.05	229839	74	0.03	1.61
	Canada	64455	48	0.07	66974	22	0.03	2.27
	Mexico	212606	4605	2.17	217113	2039	0.94	2.31
40–49	Italy	203639	531	0.26	231251	230	0.1	2.62
	Denmark	8750	*N/A	*N/A	9765	*N/A	*N/A	*N/A
	England	249577	1018	0.41	294308	637	0.22	1.88
	Israel	25618	20	0.08	28044	22	0.08	1
	Spain	244110	420	0.17	277509	219	0.08	2.18
	Canada	57065	101	0.18	63694	61	0.10	1.85
	Mexico	202369	12882	6.37	206134	5826	2.83	2.25
50–59	Italy	234433	2203	0.94	250416	803	0.32	2.93
	Denmark	8740	18	0.21	9055	11	0.12	1.7
	England	244738	3359	1.37	282393	1927	0.68	2.01
	Israel	20005	93	0.46	20948	44	0.21	2.21
	Spain	221061	1510	0.68	248460	674	0.27	2.52
	Canada	53968	329	0.61	56998	226	0.40	1.54
	Mexico	169787	23525	13.86	168598	12767	7.57	7.83
60–69	Italy	161196	6490	4.03	144962	2279	1.57	2.56
	Denmark	4784	49	1.02	4440	34	0.77	1.34
	England	141785	7575	5.34	141420	4213	2.98	1.79
	Israel	14298	237	1.66	13151	122	0.93	1.79
	Spain	152088	4234	2.78	151938	1666	1.10	2.54
	Canada	36333	1006	2.77	33915	604	1.78	1.55
	Mexico	110557	30011	27.15	99277	18672	18.81	1.44
70–79	Italy	118023	15389	13.04	110632	7137	6.45	2.02
	Denmark	2797	192	6.86	2723	75	2.75	2.49
	England	82610	16711	20.23	80328	10425	12.98	1.56
	Israel	6771	474	7.00	6496	228	3.51	1.99
	Spain	100566	9368	9.32	104748	4734	4.52	2.06
	Canada	20220	2379	11.77	20733	1644	7.93	1.48
	Mexico	60437	24188	40.02	50052	15607	31.18	1.28
80+	Italy	94917	27478	28.95	170243	30286	17.79	1.63
	Denmark	1339	281	20.99	1963	298	15.18	1.38
	England	80381	35191	43.78	125379	36573	29.17	1.50
	Israel	2975	787	26.45	4667	803	17.21	1.54
	Spain	87861	20364	23.18	156331	22388	14.32	1.62
	Canada	20482	6411	31.30	39381	8366	21.24	1.47
	Mexico	25855	12560	48.58	22457	8533	38.00	1.28

CFR = case-fatality rates, CFRR = male to female CFR ratio

*N/A—The data for Denmark were excluded since there were no deaths recorded

**Detailed disaggregated data for ages 0–9 and 10–19 in Canada were missing

**Fig 2:**
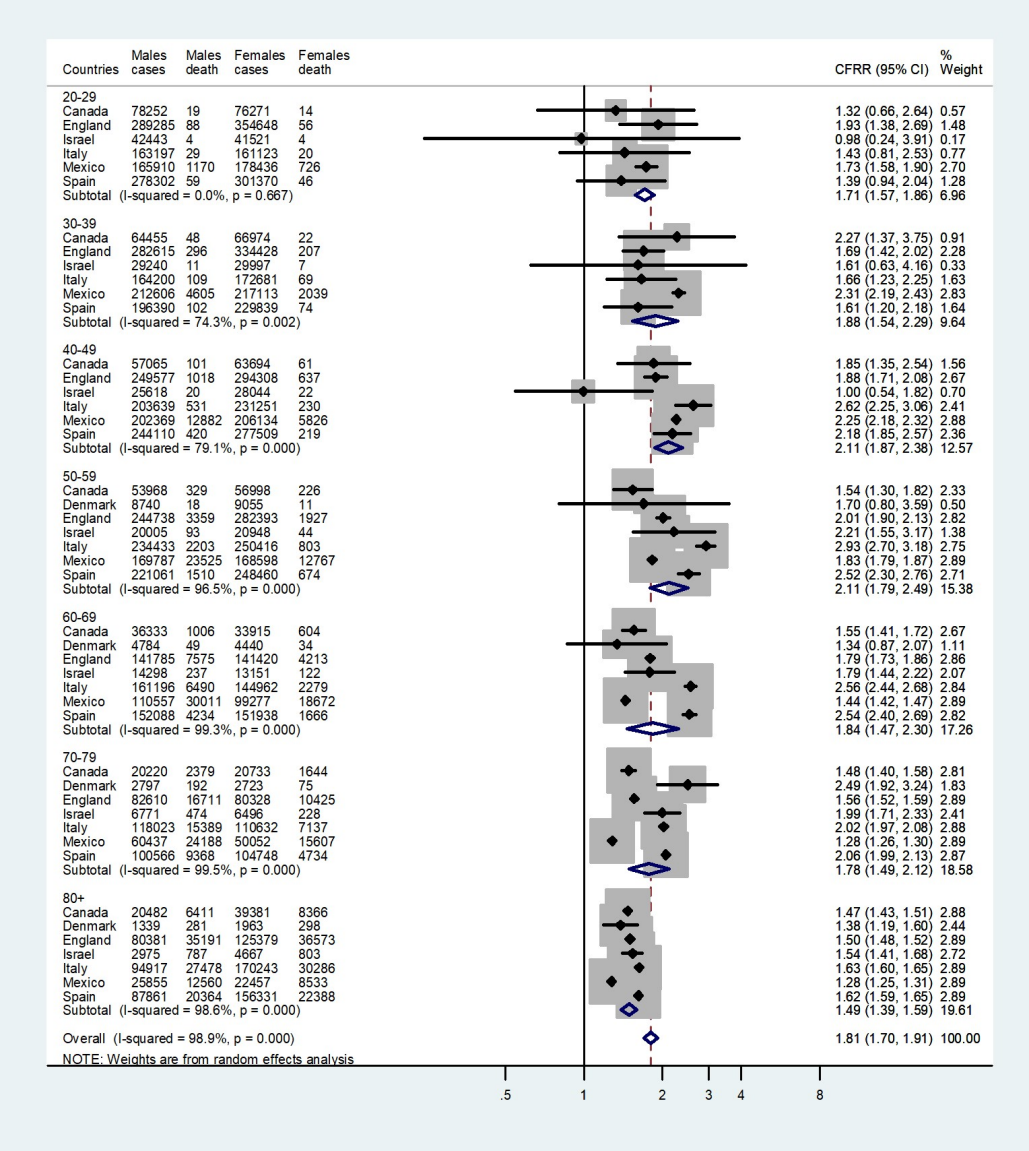
Forest plot of the ratio of the male to female CFRs (CFRR) in seven countries for each age group.

The meta-analysis of the male to female CFR ratios is shown in the forest plot by age group and country in Fig 2. The number of deaths in the age groups 0–9 and 10–19 were less than 10 in all countries except England and Mexico. Therefore, the estimates in those age groups are very unstable and were not included in the meta-analysis.

Results for each age group and country are displayed as a square and a horizontal line, representing the effect estimate together with its confidence interval. The area of the square reflects the weight that the age and country contribute to the overall study. The combined effect estimate (with confidence interval) is represented by a diamond. For all age groups and countries, the CFRs were consistently higher among males compared with females with the CFRRs ranging mostly between 1.5 and 2.5.

**Fig 3:**
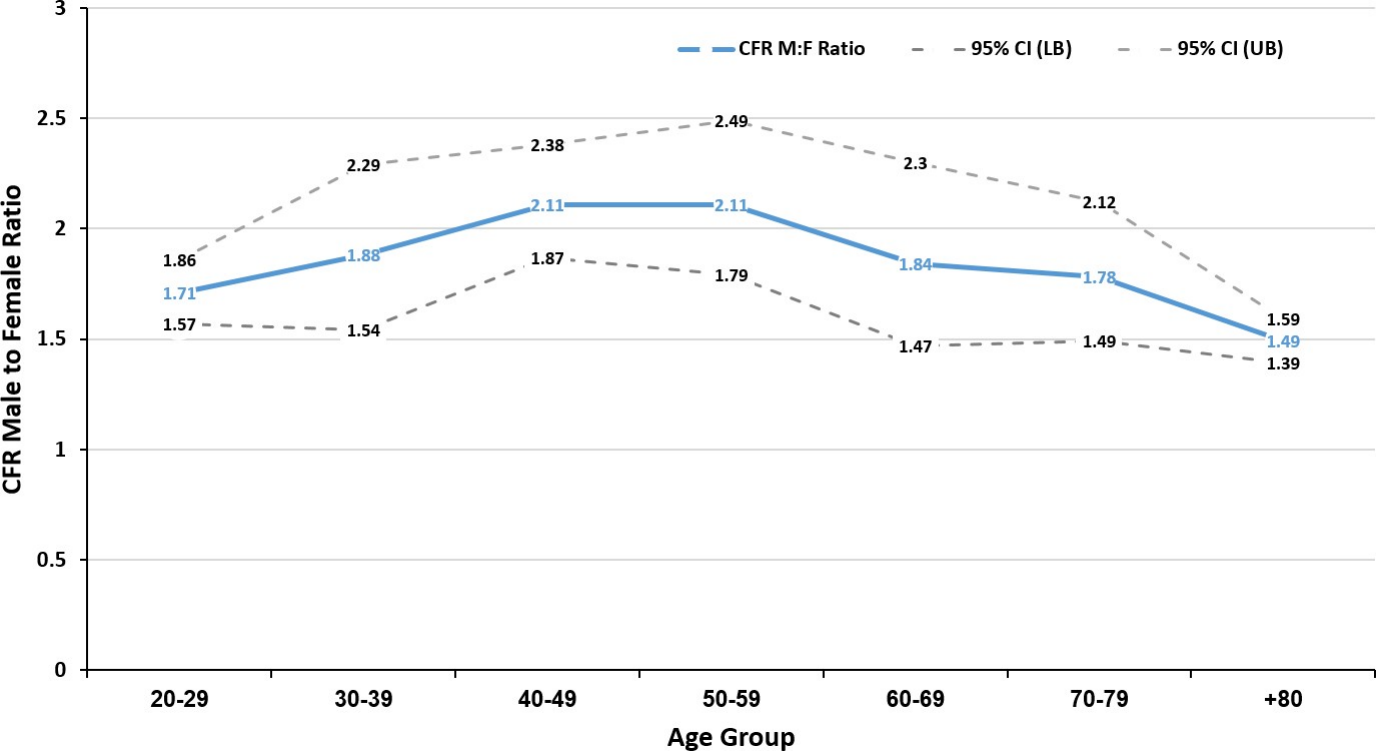
The pooled male to female CFRR (for seven countries) by age group together with the 95% CI lower and upper limits, derived from the meta-analysis.

The age-related trend in the male to female CFRRs is shown in Fig 3. Generally, the CFRR increases up to age 59, and then declines to the lowest at age 80+.

In age groups 40–60, the male CFRs are more than double those of the females, and over the age of 60, the male CFRs were 49% to 84% higher. We also looked for possible trends in male to female CFRR ratio by comparing the current updated data with that available on average three months ago (data not shown). There were moderate changes with smaller differences in the CFRRs between the age groups. However, there was no evidence of a trend in the CFRRs and the overall findings were similar.

### Sensitivity analysis

To evaluate the effect of individual countries and years on the pooled CFRRs, we performed leave-one-out sensitivity analysis and recomputed the pooled CFRRs. No significant differences were found after omitting one country at a time (Table 2).

**Table 2:** Sensitivity analysis of the pooled CFRRs for each age group, by removing one country at a time.

Country removed	Age groups						
	20–29	30–39	40–49	50–59	60–69	70–79	80+
**Canada**	1.72 (1.57–1.87)	1.83 (1.47–2.28)	2.14 (1.88–2.43)	2.24 (1.87–2.68)	1.89 (1.47–2.43)	1.84 (1.51–2.24)	1.49 (1.38–1.61)
**Denmark**	-	-	-	2.13 (1.8–2.53)	1.9 (1.5–2.41)	1.7 (1.41–2.05)	1.5 (1.4–1.61)
**England**	1.69 (1.55–1.85)	1.94 (1.57–2.41)	2.19 (1.92–2.5)	2.12 (1.67–2.69)	1.83 (1.35–2.49)	1.83 (1.44–2.32)	1.49 (1.35–1.63)
**Israel**	1.71 (1.57–1.86)	1.89 (1.54–2.32)	2.17 (1.95–2.42)	2.1 (1.76–2.51)	1.84 (1.45–2.35)	1.75 (1.44–2.11)	1.48 (1.38–1.59)
**Italy**	1.72 (1.57–1.87)	1.92 (1.55–2.39)	2 (1.75–2.3)	1.98 (1.74–2.24)	1.74 (1.41–2.13)	1.73 (1.45–2.06)	1.46 (1.36–1.58)
**Mexico**	1.58 (1.27–1.96)	1.7 (1.49–1.94)	2.02 (1.67–2.44)	2.17 (1.78–2.66)	1.94 (1.6–2.35)	1.87 (1.62–2.15)	1.54 (1.47–1.61)
**Spain**	1.73 (1.58–1.88)	1.94 (1.57–2.4)	2.08 (1.79–2.41)	2.04 (1.7–2.44)	1.74 (1.39–2.17)	1.73 (1.44–2.07)	1.47 (1.36–1.58)
**All countries together**	1.71 (1.57–1.86)	1.88 (1.54–2.29)	2.11 (1.87–2.38)	2.11 (1.79–2.49)	1.84 (1.47–2.3)	1.78 (1.49–2.12)	1.49 (1.39–1.59)

CFRR = case-fatality rate ratio

CI = confidence interval

Similar results were obtained after omission of another age group at a time (Table 3). Thus, no single country or age group substantially influenced the pooled CFRRs. This confirms that the results of this study are stable and robust.

**Table 3:** Sensitivity analysis of the pooled CFRRs for each country by removing each age group at a time.

Excluding age group	Country						
	Canada	Denmark	England	Israel	Italy	Mexico	Spain
**20–29**	1.49 (1.45–1.52)	-	1.73 (1.58–1.88)	1.69 (1.57–1.81)	2.2 (1.82–2.67)	1.68 (1.41–2)	2.08 (1.72–2.5)
**30–39**	1.49 (1.45–1.52)	-	1.74 (1.59–1.9)	1.68 (1.57–1.81)	2.21 (1.82–2.7)	1.6 (1.36–1.89)	2.05 (1.7–2.48)
**40–49**	1.49 (1.45–1.52)	-	1.71 (1.56–1.87)	1.7 (1.58–1.82)	2.07 (1.69–2.52)	1.61 (1.39–1.86)	1.97 (1.63–2.39)
**50–59**	1.49 (1.45–1.52)	1.68 (1.1–2.56)	1.67 (1.55–1.8)	1.66 (1.55–1.78)	2.02 (1.68–2.43)	1.66 (1.4–1.98)	1.92 (1.59–2.31)
**60–69**	1.48 (1.45–1.52)	1.8 (1.12–2.9)	1.72 (1.58–1.87)	1.67 (1.55–1.8)	2.08 (1.73–2.49)	1.73 (1.4–2.14)	1.92 (1.62–2.27)
**70–79**	1.49 (1.45–1.53)	1.39 (1.21–1.59)	1.78 (1.56–2.04)	1.6 (1.47–1.72)	2.13 (1.59–2.84)	1.77 (1.47–2.12)	1.96 (1.53–2.53)
**80+**	1.52 (1.45–1.59)	1.85 (1.18–2.9)	1.79 (1.61–2)	1.89 (1.69–2.12)	2.29 (1.94–2.72)	1.77 (1.47–2.12)	2.15 (1.89–2.45)
**Original country CFRR [95%CI]**	1.49 (1.45–1.52)	1.68 (1.17–2.42)	1.73 (1.59–1.89)	1.68 (1.57–1.8)	2.14 (1.78–2.58)	1.69 (1.43–1.98)	2 (1.68–2.39)

CFRR = case-fatality rate ratio

CI = confidence interval

In the meta-regression analysis, CFRR was the dependent variable and age group and country were the independent variables. The results revealed that both variables were statistically significant (age group p = 0.002 and country p = 0.025). This indicated that both the countries and the age groups contributed to the variability in the findings. However, as mentioned earlier, the higher CFR’s in males than females were consistently present in all age groups and countries.

## Discussion

Based on a comparative study of national data from seven countries, we found that the CFRs for COVID-19 were higher in males in all age groups and the sex ratio of the CFRs varied from 1.49 higher in males in the age 80+, to 2.11 in the age 40–49 and 50–59. Although the CFRR’s varied between countries, the excess CFR in males in each age group was a consistent finding with comparable magnitude. Our findings provide evidence of a large and consistent excess CFR in males for COVID-19, for all age groups, over seven countries. This study supports and extends the findings in other studies [6, 7, 24].

A major strength of the study is that it is based on data from countries with large populations and large numbers of deaths and cases. Selection bias has been minimized by using national data, which should be representative of each country. The larger number of female cases in many countries may reflect more exposure in the workplace [2–4]. The inclusion of seven countries, from different geographic location allowed us to evaluate the consistency of the findings over different populations. It does not seem likely that excluding countries that have poor diagnostic facilities or reporting has created an important source of selection bias which would influence the male to female CFRR and affect generalizability of the findings [25]. However, we cannot exclude the possibility that there could be different results from low-income countries, where detailed data are difficult to access.

There are several possible sources of bias in the study. Information bias can be present in both the numerator and denominator of the CFRs. If only those with more severe symptoms are tested this will affect the denominator of the CFR and will depend on the testing strategy of each country. For example, if tests are restricted to the more severe cases and if females tend to have a less severe disease, this would selectively underestimate the number in the CFR denominator and inflate the female CFR [26]. In such a case the male excess in CFR would be underestimated. There may be differences in the policy of performing tests and determining cause of death. However, it is unlikely differ between males and females. There may be variability in the coding of causes of death, particularly in people over 80 with multiple co-morbidities. This could differ between the sexes due to differences in comorbidities. Deaths may be underestimated because of a lack of testing after death and in some countries and it is possible that some COVID-19 deaths occurring out of hospital may be missed. However, this should not affect the male to female CFRR. The findings are strengthened by data from the Central Bureau of Statistics in Israel, demonstrating that during 2020 there was more excess mortality in men than in women (personal communication—unpublished data). Although data are lacking for the infection fatality rate (IFR), any sex differences in the IFRs should largely be reflected in the male to female CFRR. Finally, there is a lag time in the occurrence of the deaths relative to the number of cases reported, so the actual CFRs should be based on the cases reported about two weeks earlier. These data are generally not available and we do not believe that they would have any substantial effect on the CFRRs [27].

Sex differences in mortality rates from other infectious diseases have been reported. A male excess in the case-fatality rates was observed in patients diagnosed with MERS in South Korea [28]. In China, the CFR for SARS-Cov-1 in 2002–3 was higher in males [29]. In the United States, infectious disease mortality, prior to the HIV/AIDS epidemic, was more than 50% higher in males [15]. Most of the infectious disease mortality was from lower respiratory tract infections. Other infectious diseases for which a male excess in CFRs include HIV/AIDS during the post-treatment era [30], leptospirosis [31], invasive pneumococcal disease [32], and in HIV patients suffering from tuberculosis [33]. In addition, mortality rates from sepsis appear to be lower in females [34] and there appears to be a more efficient immune response to gram-negative bacteria in female mice [35]. On the other hand, for measles there appears to be excess female mortality under the age of 50 [36] and higher case-fatality rates were observed in females in cases of hemorrhagic fever with renal syndrome in China [37].

While this study cannot provide information on the mechanisms in the sex differences observed, it is possible to postulate some explanations. Regarding possible cultural factors, in the countries in this study, there is no evidence that the sex of the patient influenced the medical care given for COVID-19. There is evidence from some countries that women are more likely to seek medical care [38]. However, there is no evidence to suggest that in these countries, adult men are more likely than women to delay medical care for acute conditions of comparable severity. Sex differences in exposure due to behavioural factors could play a part in the incidence of the cases. However, since this study focuses on CFRs that should not influence the result. The severity of COVID-19 has been shown to be strongly associated with underlying conditions in the patients [39]. These include hypertension, diabetes, and obesity. However, in a large study in the United Kingdom, males were still at higher risk of death after controlling for co-morbidities [40].

The genetic and hormonal differences between males and females have been suggested as possible explanations for the higher case-fatality in males [6]. The SARS-CoV-2 virus uses the ACE2 receptor to enter the cells. It has been reported that circulating ACE2 levels are increased in male compared with female subjects, in patients with diabetes or cardiovascular diseases, and in prostate epithelial cells [41, 42]. The different hormonal environment could have extended pathophysiological role in SARS-CoV-2 occurrence, with testosterone, causing men to develop more serious complications related to the SARS-CoV-2 infection [43]. In general, for SARS-CoV, it has been reported, that estrogen signaling in females may directly suppress SARS-CoV replication via effects on cellular metabolism [44].

Estradiol promotes innate immune signaling pathways, and enhances production of pro-inflammatory cytokines and chemokines, a phenomenon that may explain the superior immune response to infection in pre-menopausal females [45–47]. In COVID-19 infected women, the production of inflammatory IL-6 (one of the main components of cytokine storm) is lower than in males and is often correlated with a better longevity [48]. Testosterone has the effect of depressing the innate and adaptive immune response [11, 49]. Thus, it is conceivable that sex hormones are implicated in the mechanism of infection by COVID-19.

However, sex hormone involvement in ACE2 regulation is likely to be important under the age of 50, when differences in hormone levels between men and women are large [50–52]. At older ages, after the age of 50–60, hormone differences probably have less significance because of profound changes in the hormonal milieu in both women and men. Although estrogen concentrations in men are about 200 times lower than that of testosterone, over the age of 50, they are higher than the concentrations in postmenopausal women [53]. In this study, the male excess in CFR’s was evident at all ages, but somewhat lower with increasing age. This could be due to a reduced effect of the differences in sex hormones.

The severity of SARS-CoV-2 appears to depend on the interaction between the virus and the individual’s immune system differentially by sex and age [54]. Genetic factors could play a part through an interaction with sex hormones [55]. The ACE2 and Ang-II receptor type 2 gene are both located on the X-chromosome and this may impact male susceptibility to COVID-19. X-chromosome genes could encourage mosaic advantage in females and sexual dimorphism that might mitigate viral infection and inflammation due to cytokine storms [56]. At older ages, genetic factors could be more dominant. With ageing, sex chromosomes undergo changes that influence their possible contribution to risk for diseases [57].

## Conclusions

In conclusion, the remarkably consistent excess COVID-19 CFRs in males in a number of countries and in all age groups, suggests that sex-specific factors influence the severity of COVID-19. The higher CFRs in males in the younger age groups could be related to a hormonal factor. These findings should stimulate research on sex as a biological variable in the pathogenesis of COVID-19.
